# Status of the Rollout of the Meningococcal Serogroup A Conjugate Vaccine in African Meningitis Belt Countries in 2018

**DOI:** 10.1093/infdis/jiz336

**Published:** 2019-10-31

**Authors:** Ado Bwaka, André Bita, Clément Lingani, Katya Fernandez, Antoine Durupt, Jason M Mwenda, Richard Mihigo, Mamoudou H Djingarey, Olivier Ronveaux, Marie-Pierre Preziosi

**Affiliations:** 1 World Health Organization (WHO) Inter-Country Support Team West Africa, Ouagadougou, Burkina Faso; 2 WHO Infectious Hazard Management, Geneva, Switzerland; 3 WHO Initiative for Vaccine Research, Geneva, Switzerland; 4 WHO Regional Office for Africa, Brazzaville, Congo; 5 WHO Infectious Hazard Management, Regional Office for Africa, Brazzaville, Congo

**Keywords:** African meningitis belt, MenAfriVac, meningococcal serogroup A, rollout

## Abstract

**Background:**

A novel meningococcal serogroup A conjugate vaccine (MACV [MenAfriVac]) was developed as part of efforts to prevent frequent meningitis outbreaks in the African meningitis belt. The MACV was first used widely and with great success, beginning in December 2010, during initial deployment in Burkina Faso, Mali, and Niger. Since then, MACV rollout has continued in other countries in the meningitis belt through mass preventive campaigns and, more recently, introduction into routine childhood immunization programs associated with extended catch-up vaccinations.

**Methods:**

We reviewed country reports on MACV campaigns and routine immunization data reported to the World Health Organization (WHO) Regional Office for Africa from 2010 to 2018, as well as country plans for MACV introduction into routine immunization programs.

**Results:**

By the end of 2018, 304 894 726 persons in 22 of 26 meningitis belt countries had received MACV through mass preventive campaigns targeting individuals aged 1–29 years. Eight of these countries have introduced MACV into their national routine immunization programs, including 7 with catch-up vaccinations for birth cohorts born after the initial rollout. The Central African Republic introduced MACV into its routine immunization program immediately after the mass 1- to 29-year-old vaccinations in 2017 so no catch-up was needed.

**Conclusions:**

From 2010 to 2018, successful rollout of MACV has been recorded in 22 countries through mass preventive campaigns followed by introduction into routine immunization programs in 8 of these countries. Efforts continue to complete MACV introduction in the remaining meningitis belt countries to ensure long-term herd protection.

Historically, large recurring meningitis epidemics affected an extensive region of sub-Saharan Africa known as the meningitis belt, which comprises whole or part of 26 countries from Senegal in the west to Ethiopia in the east. In this geographical area, outbreaks occur during the dry epidemic season, usually covering the first half of the year. The main pathogen responsible for epidemic bacterial meningitis in this region was *Neisseria meningitidis* serogroup A (NmA).

Starting in 2010, a novel meningococcal serogroup A conjugate vaccine ([MACV] the product is manufactured by a single manufacturer and is marketed under the name MenAfriVac) was prequalified by the World Health Organization (WHO) for protection against meningitis caused by NmA [1–3]. The MACV was first used widely with great acceptance and success, in December 2010, during mass vaccination campaigns targeting persons aged 1–29 years in Burkina Faso, Mali, and Niger. It was the first time that a new WHO prequalified vaccine was used in the African region with no time lag after completion of clinical trials and licensure and directly through a mass vaccination campaign rather than in routine immunization as had been done previously with new vaccines. The campaign was conducted in 1 phase in 2010 in Burkina Faso, and in 2 phases in Mali and Niger between 2010 and 2011. The MACV was then progressively introduced, into the epidemic-prone areas in other countries of the African meningitis belt, through mass preventive vaccination campaigns.

To sustain population-level immunity, WHO recommends that MACV be introduced into the routine childhood immunization program no more than 5 years after completion of mass preventive campaigns. A single dose should be administered to children aged 9–18 months. In addition, routine introduction should be accompanied by a one-time catch-up campaign for birth cohorts born since the mass campaign [4]. The Regional Strategic Plan for Immunization 2014–2020 in the WHO African Region set a target (1) for all countries within the meningitis belt to introduce MACV through mass campaigns and (2) for 15 of these countries to have introduced MACV into their routine immunization schedules by 2020 [5]. This article describes the status of MACV rollout in the meningitis belt countries and highlights the challenges that may be associated with the delay of the vaccine rollout in some countries.

## METHODS

### Country Risk Prioritization

The District Prioritization Tool (DPT) [6] is a comprehensive and standardized easy-to-use tool that has been developed for meningitis risk assessment in countries of the African meningitis belt before introduction of MACV. The DPT was used to rank countries for purposes of planning for MACV mass campaigns, as well as for future introduction into routine immunization programs. This methodology was developed for sequential country introductions based on epidemic risk and disease burden as well as the ability of countries to conduct vaccination campaigns. Using this tool, countries were ranked into 5 risk categories: Group 1 - countries with high epidemic risk and high disease burden (Burkina Faso, Chad, Ethiopia, Mali, Niger, Nigeria, and Sudan); Group 2 - countries with high epidemic risk but low disease burden (Benin, Cameroon, and Ghana); Group 3 - countries with low epidemic risk but high disease burden (Democratic Republic of the Congo and South Sudan); Group 4 - countries with an intermediate epidemic risk and disease burden (Côte d’Ivoire, Guinea, Senegal, Togo, and Uganda); Group 5 - countries with low epidemic risk and low disease burden (Burundi, Central African Republic, Eritrea, Gambia, Guinea-Bissau, Kenya, Mauritania, Rwanda, and United Republic of Tanzania).

### Resource Mobilization and Coordination

Gavi, the Vaccine Alliance, provided support for operational and vaccine costs based on the country requests for campaigns (up to $0.65 per person) and also provided cofinancing to help governments with routine immunization. Governments and other partners, such as the WHO, UNICEF, and the International Committee of the Red Cross, provided additional resources for assessment, social mobilization, and transport to reach remote areas.

The international coordination for MACV rollout was led by the WHO Regional Office for Africa and the Inter-Country Support Team Office for West Africa, based in Ouagadougou, Burkina Faso. Teleconferences were organized with implementing countries and partners (WHO, UNICEF, US Centers for Disease Control and Prevention, and Gavi) to review status of preparedness plans, monitor progress, identify constraints, and share best practices. At the country level, coordination mechanisms were established at national, regional/provincial/state, and district levels. Various working groups for training, vaccination, pharmacovigilance, logistics, monitoring and evaluation, and social mobilization were convened during the planning and operational phases.

In the early 2010s, only 2 countries in the meningitis belt were, at the time, equipped with a National Immunization Technical Advisory Group (NITAG). The NITAGs that were established in additional countries, mostly from 2013 on, have gradually became functional and provided recommendations relating to MACV rollout in time to inform routine introduction and catch-up campaigns [7].

### Vaccine Handling

The MACV received regulatory approval to be kept outside the cold chain for up to 4 days at up to 40°C in a controlled temperature chain (CTC). The CTC approach was first used on a large scale in 2014 in a vaccination campaign in 10 health districts in Togo. A survey carried out in Togo showed that the use of this approach did not have a negative impact on the vaccination coverage, and there were no significant differences in coverage in districts with CTC or in districts without CTC [8]. The CTC approach was also used in selected districts in Benin, Democratic Republic of the Congo, Côte d’Ivoire, Mauritania, and South Sudan.

Furthermore, MACV was administered to pregnant and lactating women despite the “label caution statement”. This was supported by a WHO recommendation based on a risk-benefit assessment. There were no severe adverse effects following immunization reported in this population compared with other vaccinated individuals. Furthermore, a formal evaluation, commissioned by WHO, revealed no evidence of any safety concerns when the vaccine was used in pregnant women.

### Data Collection

We reviewed country reports on MACV campaigns and routine immunization data reported to the WHO Regional Office for Africa from 2010 to 2018. We also reviewed the country plans for MACV introduction into routine immunization programs. Discussions were undertaken with country stakeholders on the likelihood of countries adhering to the period of introduction as provided in the country plans.

## RESULTS

### Meningococcal Serogroup A Conjugate Vaccine Mass Vaccination Campaigns

**Figure 1.**
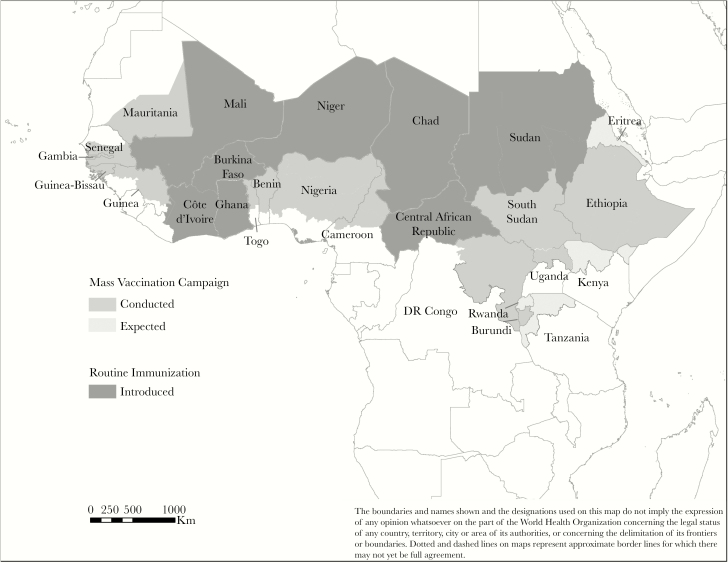
Meningococcal A Conjugate Vaccine introduction in countries of the African meningitis belt, 2010–2018.

By the end of 2018, a total of 22 of the 26 countries of the meningitis belt had introduced MACV through mass preventive campaigns targeting persons aged 1–29 years (Table 1 and Figure 1). A cumulative total of 286 882 970 eligible people received MACV via these mass vaccination campaigns (Table 2). Most of the vaccination campaigns were completed in the fourth quarter of each calendar year, before the onset of the meningitis season. All 12 countries in the risk categories 1, 2, and 3 conducted mass campaigns in the early phases of MACV rollout from 2010 to 2015. In 11 of the 22 countries that had completed campaigns by 2018, all persons aged 1–29 years had been targeted for vaccination nationwide. In the remaining 11 countries, vaccination was completed in selected high-risk districts. Four of the meningitis belt countries in risk category 5 (Eritrea, Kenya, Rwanda, and United Republic of Tanzania) have yet to conduct mass campaigns with MACV; however, 2 of them have planned campaigns for 2019. For the 2 remaining countries, gaps were observed in epidemiological data to inform the risk-assessment process, and national authorities chose to strengthen surveillance before moving to the organization of campaigns.

**Table 1.** Summary of Meningococcal Serogroup A Conjugate Vaccine Campaigns and Routine Introductions, as of December 31, 2018

	Mass Campaigns (Targeting Ages 1–29 Years)			Introduction Into Routine Immunization		Catch-up Campaigns	
Countries	Date	Number of Phases	Target Areas	Date	Target Age (Months)	Date	Target Age (Years)
Burkina Faso	2010	1	Nationwide	2017 (March)	15	2016 (October)	1–6
Mali	2010–2011	2	Nationwide	2017 (February)	9	2017 (June)	1–5
Niger	2010–2011	2	Nationwide	2017 (October)	9	Planned Q1-2019	
Chad	2011–2012	4	Nationwide	2017 (July)	9	November–December 2018	N/A
Cameroon	2011–2013	3	High risk	N/A	N/A	N/A	N/A
Nigeria	2011–2014	4	High risk	N/A	N/A	N/A	N/A
Benin	2012	1	High risk	N/A	N/A	N/A	N/A
Ghana	2012	1	High risk	2016 (November)	18	2016 (July)	1–4
Senegal	2012	1	High risk	N/A	N/A	N/A	N/A
Sudan	2012–2013	2	Nationwide	2016 (July)	9	2016 (November)	1–5
Gambia	2013	1	Nationwide	2019	18	N/A	N/A
Ethiopia	2013–2015	3	Nationwide	N/A	N/A	N/A	N/A
Cote d’Ivoire	2014	1	High risk	2018 (August)	9	2018 (December)	1–4
Mauritania	2014	1	High risk	N/A	N/A	N/A	N/A
Guinea	2015	1	High risk	N/A	N/A	N/A	N/A
Togo	2015	1	High risk	N/A	N/A	N/A	N/A
Democratic Republic of Congo	2016	1	High risk	N/A	N/A	N/A	N/A
Guinea-Bissau	2016	1	Nationwide	N/A	N/A	N/A	N/A
South Sudan	2016–2018	2	Nationwide	N/A	N/A	N/A	N/A
Central African Republic	2017	1	Nationwide	2017 (June)	9	NA	NA
Uganda	2017	1	High risk	N/A	N/A	N/A	N/A
Burundi	2018	1	Nationwide	N/A	N/A	N/A	N/A

Abbreviations: MACV, meningococcal A conjugate vaccine; N/A, not applicable.

NOTE: Four meningitis belt countries—Eritrea, Kenya, Rwanda, and Tanzania—have yet to conduct MACV mass campaigns.

**Table 2.** Population Vaccinated and Coverage Achieved With Meningococcal Serogroup A Conjugate Vaccine Preventive Mass Vaccination Campaigns and Catch-up Campaigns, 2010–2018

Countries	Regions, States, Districts Immunized	Year of Mass Campaigns	Target Population (Ages 1–29 Years)	No. Immunized	Administrative Coverage, %	Coverage via Survey, %
Benin	5 northern regions	2012	25 956 654	2 718 459	104.7	96.0
Burkina Faso	Countrywide	2010	11 133 831	11 425 391	102.6	95.9
		2016^a^	3 956 618	4 152 737	105.0	97.0
Burundi	Countrywide	2018	7 867 785	7 968 553	101.3	Ongoing
Cameroon	4 northern regions	2011–2013	6 727 388	6 725 245	100.0	73.5
Central African Republic	Countrywide	2017	3 658 248	3 220 358	88.0	93.6
Chad	Countrywide	2011–2012	9 223 913	8 732 251	94.7	Not conducted
	9 Regions	2018^a^	3 352 986	3 496 869	104.3	Not conducted
Cote d’Ivoire	25 northern districts	2014	4 271 669	4 587 056	107.4	Not conducted
	25 northern districts	2018^a^	894 645	933 070	104.3	Not conducted
Democratic Republic of Congo	6 provinces	2016	18 205 784	18 058 535	99.2	Not conducted
Ethiopia	Countrywide	2013–2015	61 748 268	61 059 389	98.9	Not conducted
Gambia	Countrywide	2013	1 177 923	1 229 509	104.4	96.6
Ghana	3 northern regions	2012	3 098 348	3 038 393	98.1	90.1
		2016^a^	679 508	666 688	98.1	96.6
Guinea	17 districts	2014–2015	3 005 423	2 880 334	95.8	92.7
Guinea Bissau	Countrywide	2016	1 277 088	1 150 136	90.1	Not conducted
Mali	Countrywide	2010–2012	10 854 599	11 109 484	102.3	96
		2016^a^	3 328 000	3 483 991	104.7	Not conducted
Mauritania	33 southern districts	2014	1 610 523	1 561 720	97.0	Not conducted
Niger	Countrywide	2010–2012	10 870 817	10 575 365	97.3	91.0
Nigeria	17 northern states	2011–2014	83 695 197	87 062 324	104.0	69.9
Senegal	8 northern regions	2012, 2014	4 383 255	4 216 691	96.2	96.2
South Sudan	6 States	2016	4 372 696	4 023 659	92.0	Not conducted
	4 States	2018	2 765 296	1 784 766	64.5	Not conducted
Sudan	Countrywide	2012–2013	24 823 460	23 521 440	94.8	94.0
		2016^a^	5 226 139	5 278 401	101.0	Not conducted
Togo	42 northern districts	2014	2 754 189	2 815 354	102.2	98.1
	1 northern district	2017	152 627	152 627	100.0	Not conducted
Uganda	39 districts	2017	6 899 267	726 5931	105.3	89.0

^a^Catch-up campaign.

The regional aggregate administrative coverage via mass campaigns in the 22 countries was 98% (range, 65%–105%) (Table 2). More rigorous coverage surveys [9–11] were conducted in 14 countries, and these confirmed that coverage among persons aged 1–29 years was >90% in 12 countries but was lower in Cameroon (74%) and Nigeria (70%) [12].

### Meningococcal Serogroup A Conjugate Vaccine Introduction Into Routine Immunization

A total of 8 of the 26 meningitis belt countries had additionally introduced MACV into their routine immunization programs as of the end of 2018: Ghana and Sudan in 2016; Burkina Faso, Central African Republic, Chad, Mali, and Niger in 2017; and Côte d’Ivoire in 2018 (Table 1). The Central African Republic introduced MACV into its routine immunization program in 2017, 2 months after the initial mass campaign so no catch-up campaign was needed [13]. In addition, Gambia plans a catch-up campaign in March 2019 before the introduction into routine immunization. Nigeria also plans MACV introduction into routine immunization and a catch-up campaign in the second half of 2019. Other countries have planned introductions during 2020 and 2021 but have yet to apply to Gavi for funding.

The target age for routine immunization varied by country: 9 months in Central African Republic, Chad, Côte d’Ivoire, Mali, Niger, and Sudan; 15 months in Burkina Faso; and 18 months in Ghana (Table 1). The median administrative coverage estimate for MACV in routine immunization in these 8 countries was 79% and ranged from 39% in Central African Republic to 84% in Burkina Faso (Table 3).

**Table 3.** Children Vaccinated With Meningococcal Serogroup A Conjugate Vaccine via Routine Immunization, 2016–2018

Countries	Regions, States, Districts Immunized	Age (Months)	Date 2016–2018	Target Population	No. Immunized	Cumulative Administrative Coverage, %
Burkina Faso	Countrywide	15	2017 (February–December)	635 025	507 067	79.8
			2018 (January–December)	777 158	654 756	84.2
Central Africa Republic	Countrywide	9	2017 (June–December)	91 399	35 589	39.0
Chad	Countrywide	9	2017 (July–December)	151 472	81 795	54.0
Ghana	Countrywide	18	2016 (November–December)	188 703	140 750	74.5
			2017	1 100 226	901 131	82.0
Mali	Countrywide	9	2017	756 614	552 789	73.0
Niger	Countrywide	9	2017 (October–December)	263 409	150 291	57.0
Sudan	Countrywide	9	2016 (July–December)	733 371	600 376	81.8
			2017	1 520 161	1 246 987	82.0
Cote d’Ivoire	26 districts	9	2018 (August–December)	368 994	266 350	78.8

### Meningococcal Serogroup A Conjugate Vaccine Catch-up Campaigns

Seven countries conducted catch-up campaigns (Table 1) associated with the introduction of the vaccine into routine immunization programs to protect those who were too young, or not born at the time of the initial mass campaign, and outside the age range targeted by the routine immunization program. In 2 countries (Ghana and Burkina Faso, which introduced at 18 and 15 months of age, respectively), the catch-up campaign targeting unprotected birth cohorts (1–7 years old) was conducted before introduction into routine immunization to ensure that no child was missed. Five countries that introduced at 9 months of age (Chad, Côte d’Ivoire, Mali, Niger, and Sudan) conducted their catch-up campaigns ≥3 months after routine introduction. A total of 18 011 756 additional children were vaccinated with MACV during catch-up campaigns at the end of 2018, producing a total of 304 894 726 individuals through mass vaccination or catch-up campaigns (Table 2). Due to competing priorities, Niger’s catch-up campaign was postponed multiple times and was only finally conducted in March 2019, more than a year after introduction into the routine immunization schedule. Administrative coverage for the catch-up campaigns ranged from 98% to 105% (Table 2).

## DISCUSSION

Successful rollout of MACV was recorded in 22 countries from 2010 to 2018, through mass preventive campaigns, catch-up campaigns, and introduction into routine immunization programs. Continued efforts are critical to complete MACV rollout in the remaining 4 meningitis belt countries and to continue introductions into routine immunization programs to ensure long-term herd protection. It is important to note that strong global coordination, country engagement, early and adequate microplanning, cascade training, community engagement, deployment of technical support, intensive supportive supervision, and adequate provision of vaccines and logistics have greatly contributed to the success of these campaigns [14].

### Meningococcal Serogroup A Conjugate Vaccine Impact

Since the introduction of MACV, the overall incidence of meningitis in the African meningitis belt has decreased dramatically, as has the risk of meningitis epidemics [15–17]. The NmA cases have disappeared completely in most countries, although sporadic cases have been reported in unvaccinated persons in Burkina Faso, Cameroon, Chad, Guinea, Niger, Nigeria, and Senegal, and 1 vaccine failure was documented in Burkina Faso [15–19].

After MACV mass campaigns, Burkina Faso reported a 71% decline in risk of suspected meningitis and a >99% decline in risk of confirmed NmA meningitis [15, 17]. In Chad, there was a 94% reduction in incidence of suspected meningitis in vaccinated versus unvaccinated districts and a 98% decrease in asymptomatic NmA carriage prevalence 4–6 months after the mass vaccination versus prevaccination era [20]. A study in 9 countries that carried out mass campaigns with MACV from 2010 and 2015 (Benin, Burkina Faso, Chad, Côte d’Ivoire, Ghana, Mali, Niger, Nigeria, and Togo) showed a 58% decline in incidence of suspected meningitis, >99% decline in incidence of confirmed NmA meningitis, a 60% decline in risk of epidemics, and an increase in incidence of non-NmA meningitis [17]. In countries participating in the MenAfriNet Consortium [21], bacterial meningitis epidemiology has varied widely by country, with overall NmA incidence remaining low, *N meningitidis* serogroups C (NmC) and W (NmW) causing several outbreaks, and *N meningitidis* serogroup X (NmX) increasing although not associated with outbreaks [22, 23].

Since 2011, the number of confirmed NmA cases reported through the WHO Regional Office for Africa laboratory network has been in decline, from 196 cases in 2011 to zero cases in 2018. However, the 2 most recent NmA cases were found in 2017 among unvaccinated individuals in Guinea and Nigeria, reinforcing the need to accelerate the rollout of MACV introduction into routine immunization programs to sustain long-term population-level protection against NmA.

### Remaining Challenges to Complete the Rollout

Despite success with MACV rollout, challenges to roll out the vaccine remain, both for remaining campaigns (initial and catch-up) and for routine immunization. Key challenges for successful campaigns have included the following: late distribution of communication materials; shortages of qualified health workers; cold-chain gaps; inadequate provision of vaccines and other supplies; insufficient supervision; lack of coordination; delay in disbursement of funds to the operational level; and delay or insufficient government contribution to operational costs and political insecurity [24].

Some of the challenges specifically related to MACV introduction into routine immunization include country motivation to include MACV in routine immunization, given the dramatic impact of the mass campaigns and resulting lower incidence of NmA. This challenge is magnified by competing public-health and immunization priorities, including the resurgence of non-NmA epidemics and initiatives for introduction of other new vaccines. However, modeling predicts that failure to introduce MACV into routine immunization programs may result in catastrophic resurgence of NmA disease [25], and that delaying the introduction will generate large pockets of susceptible populations. The promising prospect in the coming years, of a multivalent meningococcal conjugate vaccine [26] widely supplied and affordable, should not slow down the monovalent vaccine rollout, because a multivalent vaccine will not be available for several years, delays in vaccine development could occur, and rapid uptake is unlikely to happen immediately. Conversely, successful implementation of these programs could provide a strong foundation for the future introduction of multivalent meningococcal conjugate vaccines to protect against both NmA and non-A serogroups.

Additional challenges to MACV introduction into routine immunization include the following: the need for domestic financing at a greater level than with mass campaigns; providing multiple injections at one visit, such as measles/rubella (9 months [first dose]/15–18 months [second dose]) and yellow fever (9 months); vaccinating beyond 12 months-of-age; changing norms and behaviors among mothers, communities, and health workers; and, in some countries, prioritizing rollout in high-risk areas versus nationwide, which could lead to public perceptions of inequity.

### Remaining Challenges in Controlling Epidemic Meningitis

Control of meningitis outbreaks is faced with challenges of insufficient funding to implement epidemic preparedness and response plans, suboptimal laboratory capacity for rapid outbreak confirmation, lack of national contingency stockpiles of vaccines, scarcity of the global vaccine supply for reactive vaccination (meningococcal polysaccharide/conjugate vaccines), and unaffordability of currently available multivalent meningococcal conjugate vaccines.

In addition, the bacterial meningitis pathogen distribution in the meningitis belt has shown a changing epidemiology in the last 5 years with NmX emergence, NmC expansion (Niger, Nigeria), and pneumococcal outbreaks [22, 23, 27–29]. In 2018, the predominant bacterial meningitis pathogens reported from the meningitis belt were *Streptococcus pneumoniae* (33%), NmC (24%), NmX (23%), and NmW (5.6%) [30]. This shift in causes of meningitis etiology requires ongoing effective surveillance and laboratory confirmation [31], as well as improved vaccination strategies [26].

The delay in completing the rollout of MACV introduction in routine EPI programs may expose countries to the risk of NmA re-emergence. This risk could be further exacerbated by insufficient coverage of new cohorts through routine immunization and delay of, or inadequate coverage in, catch-up campaigns.

## CONCLUSIONS

Meningococcal meningitis epidemics remain a dramatic public-health problem in Africa. Through massive MACV rollout efforts, significant progress has been achieved in the decline in incidence of NmA meningitis and in the risk of epidemics due to NmA. However, non-NmA meningitis cases and outbreaks continue. Strengthening surveillance, improving laboratory capacity, and implementing prompt responses to outbreaks remains critical.

Strategies to eliminate meningitis epidemics include ensuring long-term protection against NmA disease through mass vaccination campaigns (herd protection) and routine immunization (long-term sustainability), enhancing outbreak response and control, strengthening surveillance and laboratory capacity, and promoting development and use of affordable multivalent conjugate vaccines. A global roadmap with the vision of defeating meningitis by 2030 [31] is being developed with the goal of eliminating bacterial meningitis epidemics, reducing cases and deaths from vaccine-preventable bacterial meningitis, reducing risk of disability, and improving quality of life after all cases of meningitis.

## Notes

***Acknowledgments.*** We thank all MenAfriNet partners and the Ministries of Health of the 26 African meningitis belt countries.

***Financial support.*** This work was funded by the MenAfriNet consortium (www.menafrinet.org) through a grant from the Bill & Melinda Gates Foundation (OPP1084298).

***Author contributions.*** All authors provided significant contributions on research protocol (research questions, title, literature review, methodology), data collection, analysis, development, and critical review of the manuscript.

***Disclaimer.*** The findings and conclusions of this report are those of the authors and do not necessarily represent the official position of the World Health Organization (WHO). The designations employed and the presentation of the material in this publication do not imply the expression of any opinion whatsoever on the part of WHO concerning the legal status of any country, territory, city or area, or of its authorities, or concerning the delimitation of its frontiers or boundaries. Dotted and dashed lines on maps represent approximate border lines for which there may not yet be full agreement. The mention of specific companies or of certain manufacturers’ products does not imply that they are endorsed or recommended by WHO in preference to others of similar nature that are not mentioned.

***Potential conflicts of interest.*** All authors reported grants paid to WHO from the Bill & Melinda Gates Foundation and from Gavi the Vaccine Alliance, during the conduct of the study for supporting the roll out of the meningococcal serogroup A conjugate vaccine. All authors have submitted the ICMJE Form for Disclosure of Potential Conflicts of interest. Conflicts that the editors consider relevant to the content of the manuscript have been disclosed.
